# SARS-CoV-2-related cardiovascular complications in the tropics

**DOI:** 10.1186/s41182-022-00474-9

**Published:** 2022-12-19

**Authors:** Josef Finsterer, Sounira Mehri

**Affiliations:** 1 Neurology and Neurophysiology Center, Postfach 20, 1180 Vienna, Austria; 2 Biochemistry Laboratory, Faculty of Medicine, LR12ES05 “Nutrition-Functional Foods and Vascular Health”, Monastir, Tunisia

**Keywords:** SARS-CoV-2, COVID-19, Arrhythmias, Heart failure, Myocarditis

## Abstract

One of the organs affected by SARS-CoV-2 is the heart. Cardiac manifestations of SARS-CoV-2 include acute coronary syndrome, endocarditis, myocarditis, pericarditis with pericardial effusion, heart failure, Takotsubo syndrome, arrhythmias, intra-ventricular thrombus formation, and cardiogenic shock. If COVID-19 patients present with cardiac complications, they require thorough cardiologic work-up, including coronary angiography if myocardial infarction or Takotsubo syndrome is suspected. Since patients with prosthetic valves and those carrying devises are prone to experience cardiac complications from a SARS-CoV-2 infection, they require particular attention and surveillance. If myocarditis is suspected, the diagnosis should be established by cardiac MRI with contrast medium or endo-myocardial biopsy.

Letter to the Editor,

We eagerly read the article by Lalani et al. about a retrospective study of 730 patients in whom COVID-19 was complicated by cardiac involvement, manifesting as acute coronary syndrome (ACS), myocarditis, cardiogenic shock, pericardial effusion, intra-cardiac thrombus formation, or as arrhythmias [1]. It was found that white blood cell count (WBC), neutrophil count, neutrophil/lymphocyte ratio (NLR), platelet/lymphocyte ratio (PLR), creatinine, D-dimer, ferritin, LDH, tachycardia, and lymphocyte count correlated with disease severity [1]. It was concluded that age, acute kidney injury, elevated WBC count, a greater PLR, low platelet count, and COVID-19 severity were independent predictors of mortality [1]. The study is appealing, but raises concerns which require discussion.

The main shortcoming of the study is its retrospective design [1]. A retrospective design does not guarantee that the investigations applied were the same for all included patients, were carried out by the same investigators, and had been standardised [1].

Another shortcoming is that myocardial infarction was defined upon clinical and ECG criteria, but not upon coronary angiography findings [1]. Diagnosing myocardial infarction solely upon clinical and ECG criteria can be misleading, and may lead to erroneous results [2]. Therefore, we should be informed about how many patients diagnosed with ST-segment elevation myocardial infarction (STEMI) and non-ST-segment elevation myocardial infarction (NSTEMI) underwent coronary angiography, how many of them underwent percutaneous transluminal angioplasty (PTA) with stent placement or atherotomy.

A further shortcoming of the study is that Takotsubo syndrome (TTS) was not considered as a cardiac complication of a SARS-CoV-2 infection. Therefore, it would be interesting to know how many of those diagnosed with STEMI or NSTEMI had undergone transthoracic echocardiography and coronary angiography to assess how often STEMI or NSTEMI was in fact a TTS. TTS mimics myocardial infarction, clinically, blood chemically, electrocardiographically, and echocardiographically, but coronary angiography should be normal according to the Mayo Clinic diagnostic criteria [3]. TTS has been repeatedly reported as a complication of a SARS-CoV-2 infection.

Another cardiac complication of a SARS-CoV-2 infection not considered in the study is endocarditis [4]. Endocarditis has been occasionally reported as a complication of SARS-CoV-2 infections [4] and there are indications that a SARS-CoV-2 infection can be a risk factor for prosthetic endocarditis [5]. Therefore, we should be informed how many of the included patients had a prosthetic valve or carried a device. Pacemakers, implantable cardioverter defibrillators, and cardiac resynchronisation therapy (CRT) system seem to carry also an increased risk of SARS-CoV-2-related endocarditis [6].

A further shortcoming of the study is that myocarditis was defined as new onset left, respectively, right systolic dysfunction together with elevated troponin [1]. Myocarditis should be diagnosed upon cardiac MRI with gadolinium or by endo-myocardial biopsy. Since either method was not applied, the diagnosis myocarditis remains questionable in the 60 patients diagnosed with myocarditis.

Overall, the interesting review has several limitations which challenge the results and their interpretation. Clarifying these weaknesses would strengthen the conclusions and could improve the study. The spectrum of cardiac disease complicating a SARS-CoV-2 infection is broader than anticipated and diagnostic procedures to detect SARS-CoV2-related cardiac disease need to be sophisticated.

## Data Availability

All data are available from the corresponding author.
